# YKL-40 protein expression in human tumor samples and human tumor cell line xenografts: implications for its use in tumor models

**DOI:** 10.1007/s13402-021-00630-z

**Published:** 2021-08-25

**Authors:** Lukas Clemens Böckelmann, Theresa Felix, Simona Calabrò, Udo Schumacher

**Affiliations:** 1 Institute of Anatomy and Experimental Morphology, Center for Experimental Medicine, University Cancer Center Hamburg, University Medical Center Hamburg-Eppendorf, Hamburg, Germany; 2 Department of Oncology, Hematology and Bone Marrow Transplantation With Section Pneumology, University Cancer Center Hamburg, University Medical Center Hamburg-Eppendorf, Hamburg, Germany

**Keywords:** Cancer, CHI3L1, Immunohistochemistry, Tissue array, Tumorigenesis, Xenograft, YKL-40

## Abstract

**Background:**

YKL-40, also known as non-enzymatic chitinase-3 like-protein-1 (CHI3L1), is a glycoprotein expressed and secreted mainly by inflammatory cells and tumor cells. Accordingly, several studies demonstrated elevated YKL-40 serum levels in cancer patients and found YKL-40 to be correlated with a poor prognosis and disease severity in some tumor entities. YKL-40 was suggested to be involved in angiogenesis and extracellular matrix remodeling. As yet, however, its precise biological function remains elusive.

**Methods:**

As YKL-40 protein expression has only been investigated in few malignancies, we employed immunohistochemical detection in a large multi-tumor tissue microarray consisting of 2,310 samples from 72 different tumor entities. In addition, YKL-40 protein expression was determined in primary mouse xenograft tumors derived from human cancer cell lines.

**Results:**

YKL-40 could be detected in almost all cancer entities and was differently expressed depending on tumor stage and subtype (e.g., thyroid cancer, colorectal cancer, gastric cancer and ovarian cancer). While YKL-40 was absent in in vitro grown human cancer cell lines, YKL-40 expression was upregulated in xenograft tumor tissues in vivo.

**Conclusions:**

These data provide new insights into YKL-40 expression at the protein level in various tumor entities and its regulation in tumor models. Our data suggest that upregulation of YKL-40 expression is a common feature in vivo and is finely regulated by tumor cell-microenvironment interactions.

**Supplementary Information:**

The online version contains supplementary material available at 10.1007/s13402-021-00630-z.

## Introduction

YKL-40, also known as chitinase-3-like protein 1 (CHI3L1) is, as its name implicates, a chitinase-like glycoprotein, but is devoid of chitinase activity caused by mutations present in its active site [1, 2]. It is secreted by a variety of cells including cancer cells, inflammatory cells such as (tumor-associated) macrophages and neutrophils, and by chondrocytes, synovial cells and smooth muscle cells [3–8]. Accordingly, elevated serum levels of YKL-40 have been found in various inflammatory and malignant diseases [9–11]. Some studies have demonstrated a positive correlation of elevated serum levels of YKL-40 and a poor prognosis in solid tumors while others presented negative results [12]. In case a correlation between YKL-40 secretion and malignant progression exists, it has been suggested that YKL-40 exerts its function mainly by stimulation of angiogenesis and regulation of extracellular matrix signaling [13–18]. A key component of this function is thought to be the ability of YKL-40 to bind to heparin and extracellular and cell surface heparan sulfate [19]. Moreover, YKL-40 is known to play a potential role in promoting tumor cell proliferation and survival and to exhibit own growth factor activity [13, 20, 21]. Studies investigating YKL-40 as a therapeutic target, however, yielded opposing results. In one study, the application of a neutralizing anti-YKL-40 antibody reduced tumor growth in a human glioblastoma (U87) xenograft model [22]. In another human melanoma xenograft (LOX) model targeting YKL-40 led to increased tumor growth within hours after injection [23].

Given the multiple and diverse functions attributed to YKL-40 and the reported opposing functions it can execute in malignant progression, its expression may differ depending on tumor entity and tumor subtype. Comprehensive protein expression data in tumor tissues, however, are lacking. Therefore, we set out to assess YKL-40 protein expression immunohistochemically in a large multi-tumor tissue microarray (TMA). In addition, we analyzed YKL-40 expression in widely used tumor cell lines taken directly from 2D cultures and from xenograft tumor tissues of these cell lines.

## Materials and methods

### Xenograft tumors of human cancer cell lines

Xenografted primary human tumors grown in immunodeficient scid mice were retrieved from the files of the Institute of Anatomy and Experimental Morphology from previous experiments. Small cell lung cancer: H69AR (RRID:CVCL_3513)*, NCI-H69 (RRID:CVCL_1579)*, NCI-H82 (RRID:CVCL_1591)*, and SW2 (RRID:CVCL_R777)* [24]; colon cancer: HT-29 (RRID:CVCL_0320)*, SW480 (RRID:CVCL_0546)*, CaCo2 (RRID:CVCL_0025)*, and HCT-116 (RRID:CVCL_0291)* [25, 26]; breast cancer: DU4475 (RRID:CVCL_1183)*, MCF-7 (RRID:CVCL_0031)*, MDA-MB-231 (RRID:CVCL_0062)*, and T-47D (RRID:CVCL_0553)* [25, 27, 28]; melanoma: FEMX-1 (RRID:CVCL_A011)*, LOX-IMVI (RRID:CVCL_1381)*, MDA-MB-435 (RRID:CVCL_0417)*, MeWo (RRID:CVCL_0445)*, and MV3 (RRID:CVCL_W280)* [27, 29]; neuroblastoma: LA-N-1 (RRID:CVCL_1827)*, LA-N-5 (RRID:CVCL_0389), SK-N-SH (RRID:CVCL_D044), IMR32 (RRID:CVCL_0346), KELLY (RRID:CVCL_2092), and LS (RRID:CVCL_2105) [30]; osteosarcoma: HOS (RRID:CVCL_0312)* and U2OS (RRID:CVCL_0042); pancreatic cancer: PaCa5072 (RRID:CVCL_C887)*, PaCa5061 (RRID:CVCL_C886)*, BxPC-3 (RRID:CVCL_0186)*, and PANC-1 (RRID:CVCL_0480)* [31]; prostate cancer: DU145 (RRID:CVCL_0105)*, LNCaP (RRID:CVCL_0395)*, PC-3 (RRID:CVCL_0035)*, and C5 [32, 33]; ovarian cancer: OVCAR3 (RRID:CVCL_0465)* and SKOV3 (RRID:CVCL_0532)* [26]; head and neck squamous cancer: UT-SCC-2 (RRID:CVCL_7820)*, UT-SCC-16A (RRID:CVCL_7812)*, UT-SCC-24A (RRID:CVCL_7826)*, UT-SCC-24B (RRID:CVCL_7827)*, UT-SCC-60A (RRID:CVCL_A089)*, and Carey24 [34]. Cell lines marked with an asterisk have been authenticated by the DSMZ-German Collection of Microorganisms and Cell Cultures GmbH, Braunschweig, Germany using short tandem repeat (STR) profiling.

### Multi-tumor tissue microarray (TMA)

For the production of TMAs, tissue cylinders with a diameter of 0.6 mm were punched from representative tumor or normal areas of each tissue block and transferred to recipient paraffin blocks. All tumor samples were obtained from the archives of the Institute of Pathology of the University Medical Center Hamburg Eppendorf. The use of archived diagnostic left-over tissues for the manufacturing of TMAs and their analysis for research purposes has been approved by local laws (HmbKHG, §12,1) and by the local ethics committee (Ethics commission Hamburg, WF-049/09). All work was carried out in compliance with the Helsinki Declaration. Freshly cut TMA sections were immunostained on one day and in one experiment (see below).

### Fixation, embedding and sectioning of cancer cells and xenograft tumors

Preparation of cancer cells as formalin-fixed paraffin-embedded (FFPE) samples was carried out as previously described [35]. Briefly, cancer cells were collected from culture flasks, fixed in formalin and next embedded into agar pellets. Agar pellets and formalin-fixed xenograft tumors were then subjected to standardized tissue infiltration using a Leica TP1020 tissue processor (Leica Biosystems, Nussloch, Germany). Subsequent paraffin embedding was performed using a Leica EG1160 Paraffin Embedding Center (Leica Biosystems, Nussloch, Germany). FFPE samples were sectioned with a thickness of 4 µm, mounted on HistoBond® glass slides (Paul Marienfeld, Lauda-Königshofen, Germany) and allowed to air-dry, followed by drying in an incubator at 37 °C overnight.

### Immunohistochemistry (IHC)

FFPE sections were de-paraffinized in two changes of xylene replacement (5 min each) and rehydrated in a series of graded ethanol (100, 96, 70 and 50% for 5 min each). Next, sections were rinsed for 5 min each with Tris-buffered saline/0.1% Tween 20 (TBS-T) and TBS (pH 7.6). The subsequent incubation steps were carried out in a moist chamber. Sections were blocked with goat serum (#X0907, Dako, Carpinteria, CA, USA; diluted 1:10) for 30 min at room temperature (RT). Directly afterwards, sections were incubated with an anti-YKL-40 primary monoclonal mouse antibody (MAb 201.F9, 2.1 mg/ml, IgG2b, epitope GAWRGTTGHHS, aa 210–220) diluted 1:100 in antibody diluent (#S0809, Dako, Carpinteria, CA, USA) for 60 min at RT. For isotype control, mouse IgG2b antibody (#16–4732-85, eBioscience, San Diego, CA, USA; diluted 1:50) was used. Staining specificity was validated by preincubation of antibody MAb 201.F9 with recombinant human YKL-40 protein (#2599-CH, R&D Systems, Minneapolis, MN, USA) for 1 h at RT to block YKL-40 binding sites and reveal possible nonspecific staining to the sections (Supplementary Fig. S2). After incubation, slides were rinsed twice with TBS-T as well as with TBS for 5 min each. Subsequently, slides were incubated with secondary biotin-conjugated goat anti-mouse antibody (#E0433, Dako, Carpinteria, CA, USA) at a dilution of 1:200 in TBS for 30 min at RT, followed by rinsing twice with TBS-T and once with TBS for 5 min each. Next, sections were treated with Vectastain® ABC-AP Kit (#AK5000, Linaris, Drossenheim, Germany) according to the manufacturer’s recommendations for 30 min at RT and again washed in TBS-T and TBS as described above. Finally, alkaline phosphatase enzyme activity was visualized by incubating the sections with Permanent Red solution (#ZUC001-125, Zytomed Systems GmbH, Berlin, Germany) for 20 min and counterstained with hematoxylin for 4 s, with intermediate washes under running tap water (3 min) and in aqua dest (2 min). Slides were dehydrated in a series of graded ethanol (70% for 15 s, 96 and 100% for 5 min each) and three changes of xylene replacement (5 min each) and, finally, covered with Eukitt® Mounting Medium (#03989, Sigma-Aldrich, Taufkirchen, Germany) and coverslips.

### Microscopy and image analysis

IHC processed sections were first evaluated using a ZEISS Axiophot 2 microscope (Carl Zeiss, Jena, Germany). Digital images were obtained using a ZEISS Axio Scan Z1 slide scanner equipped with a ZEISS EC Plan-Neofluar 20x/0,50 Pol M27 objective (Carl Zeiss, Jena, Germany) and a Hitachi HV-F20SCL camera with 1600 × 1200 pixels (Hitachi Kokusai Electric America Ltd., NY, USA). For image acquisition, ZEISS ZEN 2.3 software was used (Carl Zeiss, Jena, Germany). Images were further processed using netScope Viewer software (Net-Base Software, Freiburg, Germany).

YKL-40 staining was observed in the cytoplasm and as fine granular staining in the extracellular matrix. Staining intensity was assessed on a five-step scale (negative, weak, intermediate-low, intermediate-high, high). Further analysis included trichotomization of staining intensities (negative; weak and intermediate-low = ‘low’; intermediate-high and high = ‘high’).

## Results

### YKL-40 protein expression in primary human tumor samples

**Fig. 1:**
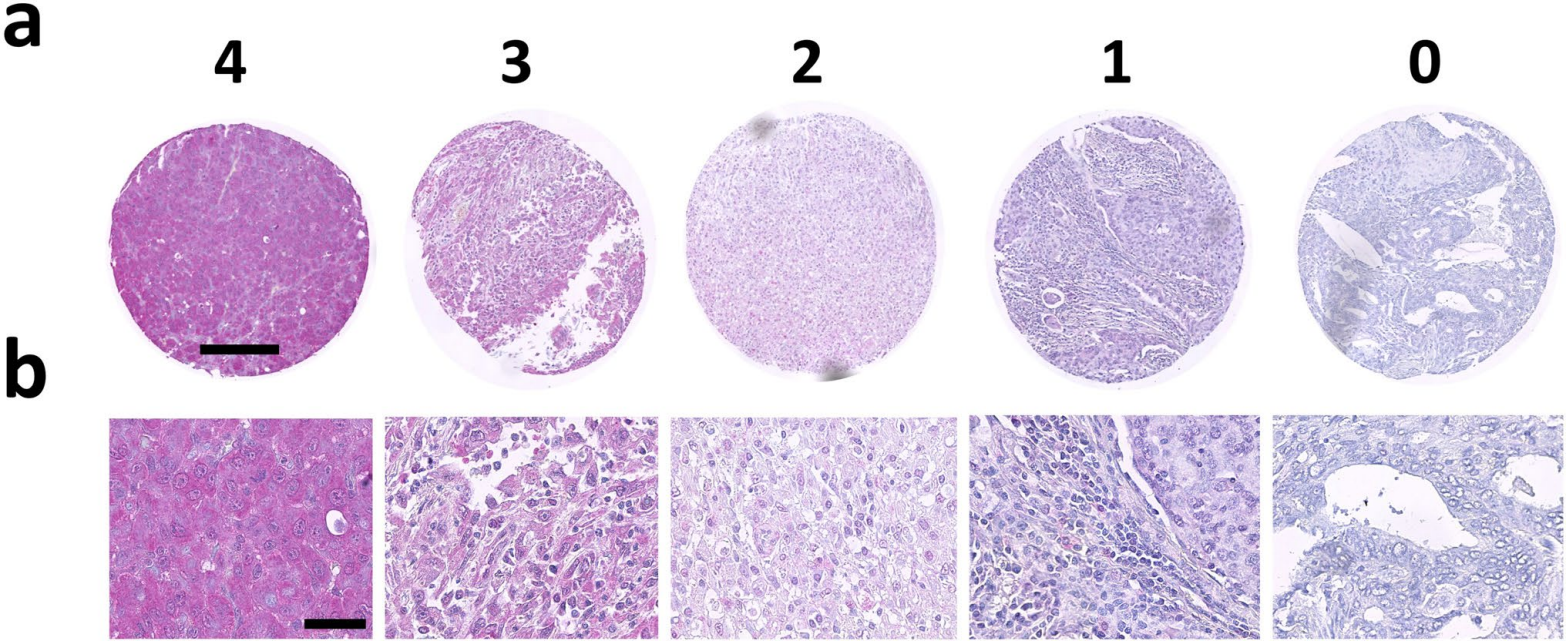
Representative images of YKL-40 immunostaining results on multi-tumor tissue microarray (TMA). Cancer cells were identified morphologically, and the staining intensities were assigned to four categories: 4 = high, 3 = intermediate-high, 2 = intermediate-low, 1 = low, and 0 = no staining. **a** Low magnification of whole tissue sample. Scale bar = 200 µm. **b** High magnification. Scale bar = 50 µm

**Fig. 2:**
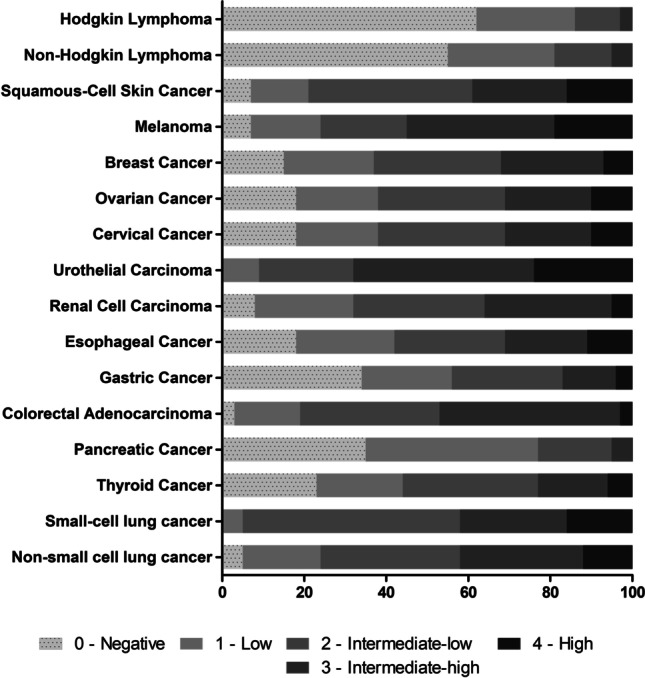
Overview of YKL-40 protein expression in selected entities on multi-tumor TMA. Where applicable, subtypes were grouped and analyzed as one (see also Table 1)

In our TMA analysis 2,310 of 3,079 tumor samples were interpretable. Non-informative cases (n = 769; 25%) were due to lack of tissue samples or absence of unequivocal cancer tissues in the respective TMA spots. In the total cohort, negative (‘0’) YKL-40 expression was found in 15.9% of all cancers, low (‘1’) in 20%, intermediate-low (‘2’) in 33.6%, intermediate-high (‘3’) in 22.2%, and high expression (‘4’) in 8.4% of the cancers (Table 1 and Fig. 2). YKL-40 protein expression was observed as a diffuse, often granular, cytoplasmatic staining with varying intensity. Cell membranes were consistently negative. In most tumors the extracellular matrix showed varying granular staining, suggesting that the cancer cells had released YKL-40. Tumor-infiltrating leukocytes residing in the adjacent tumor stroma frequently stained positive (Fig. 6d–f). The percentage of tumor samples with positive stroma cell staining and the proportion of tumor samples with staining intensity in stroma cells surpassing that in tumor cells are reported in Table 1 as well. Representative IHC images of negative, intermediate-low, intermediate-high, and strong YKL-40 protein expression are shown in Fig. 1. Anti YKL-40 monoclonal antibody Mab 201.F9 was tested to be specific for human YKL-40. Cross-reactivity with murine YKL-40 was excluded (Supplementary Fig. S1).

**Table 1:** Immunohistochemical detection of YKL-40 protein expression in a multi-tumor tissue microarray Staining intensity was assessed on a scale from ‘0’ = negative over ‘1’ = weak to ‘4’ = high expression

				YKL-40 Staining Intensity %					Matrix Cells %	
Organ system	Tissue	Specific tumor entity	n	0	1	2	3	4	Pos	> Tumor
Skin		Basalioma	41	4.9	36.6	46.3	12.2	0.0	76.2	48.8
		Benign Naevus	26	0.0	11.5	26.9	42.3	19.2	81.5	7.7
		Squamous cell carcinoma	43	7.0	14.0	39.5	23.3	16.3	55.6	14.0
		Melanoma	42	7.1	16.7	21.4	35.7	19.0	78.0	26.8
Head, Chest, and Respiratory Tract	Larynx	Squamous cell carcinoma	41	22.0	19.5	31.7	24.4	2.4	34.9	24.4
	Oral cavity	Squamous cell carcinoma	50	2.0	10.0	32.0	48.0	8.0	71.4	10.2
	Lung	Squamous cell carcinoma	33	9.1	15.2	24.2	45.5	6.1	56.3	16.7
		Bronchial carcinoma, large cell	22	9.1	18.2	31.8	18.2	22.7	72.7	28.6
		Adenocarcinoma	32	0.0	9.4	31.3	40.6	18.8	90.6	22.6
		Bronchoalveolar carcinoma	6	0.0	33.3	50.0	16.7	0.0	16.7	0.0
		Small-cell carcinoma	19	0.0	5.3	52.6	26.3	15.8	50.0	100.0
	Salivary glands	Parotis, pleomorphic adenoma	48	58.3	20.8	18.8	2.1	0.0	2.1	60.4
		Parotis, Warthin tumor	44	2.3	22.7	25.0	43.2	6.8	0.0	2.3
		Basal cell adenoma	14	7.1	28.6	35.7	28.6	0.0	21.4	7.1
Gynecologic	Vagina	Squamous cell carcinoma	24	29.2	20.8	25.0	16.7	8.3	45.8	37.5
	Vulva	Squamous cell carcinoma	33	0.0	6.1	30.3	39.4	24.2	60.0	12.1
	Cervix	Squamous cell carcinoma	37	27.0	27.0	18.9	16.2	10.8	35.1	37.8
		Adenocarcinoma	44	2.3	11.4	40.9	34.1	11.4	55.6	6.8
	Endometrium	Carcinoma, endometroid	47	55.3	27.7	17.0	0.0	0.0	4.3	59.6
		Carcinoma, serous	36	19.4	27.8	36.1	13.9	2.8	13.9	22.2
	Uterus	Stroma sarcoma	12	8.3	8.3	50.0	33.3	0.0	50.0	16.7
		Carcinosarcoma	46	4.3	32.6	39.1	21.7	2.2	45.7	13.0
	Ovaries	Carcinoma, endometroid	25	4.0	28.0	20.0	32.0	16.0	67.9	16.0
		Carcinoma, serous	39	5.1	15.4	46.2	23.1	10.3	76.2	26.3
		Carcinoma, mucinous	23	43.5	17.4	26.1	8.7	4.3	50.0	56.5
		Brenner tumor	5	80.0	0.0	20.0	0.0	0.0	66.7	80.0
	Breast	Carcinoma, ductal (NST)	30	56.7	20.0	13.3	10.0	0.0	10.0	56.7
		Carcinoma, lobular	28	7.1	25.0	46.4	21.4	0.0	63.3	25.9
		Carcinoma, medullary	11	0.0	0.0	63.6	36.4	0.0	45.5	0.0
		Carcinoma, tubular	10	0.0	20.0	20.0	40.0	20.0	83.3	20.0
		Carcinoma, mucinous	14	7.1	21.4	14.3	28.6	28.6	35.7	21.4
		Carcinoma, phyllodes	44	18.2	43.2	27.3	11.4	0.0	47.8	50.0
Gastrointestinal	Large intestine	Adenoma, low grade	17	29.4	23.5	29.4	11.8	5.9	17.6	29.4
		Adenoma, high grade	19	21.1	21.1	26.3	21.1	10.5	55.0	31.6
		Adenocarcinoma	32	3.1	15.6	34.4	43.8	3.1	56.3	12.5
	Small intestine	Adenocarcinoma	6	0.0	0.0	83.3	16.7	0.0	71.4	33.3
	Stomach	Carcinoma, diffuse type	29	65.5	27.6	6.9	0.0	0.0	37.5	86.2
		Carcinoma, intestinal type	30	3.3	16.7	46.7	26.7	6.7	74.2	20.7
	Esophagus	Adenocarcinoma	31	29.0	32.3	22.6	12.9	3.2	34.4	35.5
		Squamous cell carcinoma	26	7.7	15.4	30.8	26.9	19.2	59.3	23.1
	Anal canal	Squamous cell carcinoma	18	27.8	11.1	33.3	27.8	0.0	42.1	27.8
	Liver	Cholangiocellular carcinoma	27	11.1	22.2	18.5	18.5	29.6	71.4	22.2
		Hepatocellular carcinoma	39	2.6	7.7	38.5	41.0	10.3	39.4	6.1
	Pancreas	Adenocarcinoma, ductal	36	36.1	33.3	25.0	5.6	0.0	25.6	50.0
		Adenocarcinoma, papillary	18	33.3	50.0	11.1	5.6	0.0	16.7	44.4
		Neuroendocrine Tumor	27	7.4	18.5	40.7	18.5	14.8	57.1	14.8
		Gastrointestinal Stroma tumor	33	6.1	12.1	42.4	36.4	3.0	73.0	25.0
Urogenital	Urothelial	Carcinoma, pTa	35	0.0	8.6	20.0	42.9	28.6	80.6	3.4
		Carcinoma, pT2-4	43	0.0	9.3	25.6	44.2	20.9	97.6	23.8
	Kidney	Carcinoma, clear cell	44	4.5	18.2	43.2	31.8	2.3	54.5	11.6
		Carcinoma, papillary	34	0.0	38.2	29.4	29.4	2.9	47.2	12.1
		Carcinoma, chromophobe	43	18.6	16.3	23.3	32.6	9.3	16.3	20.9
		Oncocytoma	41	2.4	0.0	9.8	19.5	68.3	56.1	2.4
	Prostate	Adenocarcinoma	46	32.6	17.4	30.4	10.9	8.7	60.9	52.2
	Germ cell tumor	Seminoma	48	2.1	16.7	68.8	12.5	0.0	68.1	23.4
		Carcinoma, embryonal	43	4.7	18.6	58.1	18.6	0.0	55.3	26.3
		Yolk sac tumor	41	4.9	19.5	41.5	22.0	12.2	57.9	18.9
		Teratoma	29	3.4	10.3	41.4	27.6	17.2	77.4	39.3
Endocrine	Thyroid	Adenoma	42	11.9	40.5	31.0	11.9	4.8	45.2	23.8
		Papillary carcinoma	45	37.8	20.0	22.2	17.8	2.2	28.3	37.8
		Follicular carcinoma	45	4.4	26.7	42.2	17.8	8.9	47.8	11.1
		Medullary carcinoma	45	40.0	17.8	35.6	6.7	0.0	28.3	46.7
		Anaplastic carcinoma	24	8.3	20.8	33.3	25.0	12.5	37.5	20.8
	Adrenal gland	Adenoma	43	2.3	9.3	62.8	25.6	0.0	7.0	4.7
		Carcinoma	13	7.7	23.1	30.8	23.1	15.4	15.4	8.3
		Pheochromocytoma	26	19.2	23.1	42.3	15.4	0.0	20.0	20.8
		Neuroendocrine tumor	22	18.2	18.2	54.5	4.5	4.5	45.8	36.4
Hematologic		Hodgkin-Lymphoma	37	62.2	24.3	10.8	2.7	0.0	45.9	75.7
		Non-Hodgkin-Lymphoma	42	54.8	26.2	14.3	4.8	0.0	47.6	57.1
		Thymoma	26	7.7	53.8	34.6	3.8	0.0	30.8	30.8
Soft tissue		Leiomyoma	48	8,3	29.2	45.8	14.6	2.1	0.0	8.3
		Leiomyosarcoma	48	8,3	10.4	56.3	22.9	2.1	35.4	12.5

### YKL-40 is differently expressed in tumor subtypes

Previously, YKL-40 was found to be elevated in sera of various tumor patients including those carrying solid tumors, leukemias and lymphomas [36]. In our study, a striking difference in YKL-40 tissue protein expression was observed when comparing solid tumors with lymphomas. About 50% of all Hodgkin and Non-Hodgkin lymphomas did not show any YKL-40 expression using IHC (Fig. 2). This observation suggests that in lymphomas a substantial proportion of serum YKL-40 may be derived from stroma and immune cells due to the tumor’s immunologic reaction, rather than from the tumors cells themselves.

**Fig. 3:**
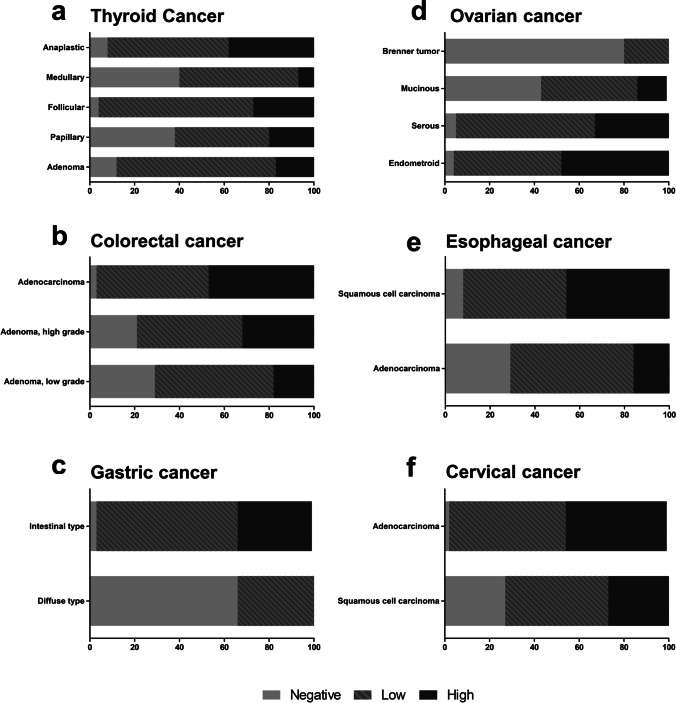
Differential expression of YKL-40 protein in selected tumor types and subtypes on multi-tumor TMA. **a** Thyroid cancer subtypes, **b** colorectal cancer, **c** gastric cancer, **d** ovarian cancer, **e** esophageal cancer and **f** cervical cancer

In solid tumors, on the other hand, tumor samples stained positive more frequently and YKL-40 was differently expressed depending on tumor subtype (Fig. 3). For further analysis, we trichotomized our results into a YKL-40 negative group, a YKL-40 low (low and intermediate-low) group and a YKL-40 high (intermediate-high and high) group to more clearly highlight those tumors and subtypes with differentially regulated YKL-40 expression. Accordingly, in the total cohort, negative YKL-40 expression was found in 15.9% of all cancers, while low YKL-40 expression was found in 53.6%, and high expression in 30.5% of all cancers. In the past, elevated serum YKL-40 levels have been proposed as prognostic biomarkers in some cancer entities. This association can be recapitulated for some entities analyzed in our TMA, while for others prognostic associations are much less suggestive. In particular, we found that in thyroid cancer, the total proportion of YKL-40-positive tumors was relatively lower in the prognostically favorable papillary and medullary carcinomas (62.2% and 60%, respectively, Fig. 3a). Contrary, in prognostically less favorable follicular carcinomas, a considerably higher percentage of YKL-40-positive tumors (95.6%) was observed. Looking at the fraction of only YKL-40 strongly expressing tumors, it was found to be highest in undifferentiated anaplastic carcinomas (37%) having a median survival of only a few months [37]. Similar, in colorectal cancer, positive YKL-40 tissue expression increased from low-grade over high-grade to adenocarcinoma (samples positive in 70.6, 78.9, and 96.9%, respectively; Fig. 3b).

In other cancer entities, YKL-40 expression was found to be associated with certain tumor subtypes. In gastric cancer, YKL-40 expression was highly associated with the intestinal type (96.7% positive), while 65.5% of tumors of the diffuse type were negative for YKL-40 expression (Fig. 3c). In serous and endometrioid differentiated ovarian cancer, YKL-40 expression was present in 95 and 96% of the tumors, respectively. On the contrary, ovarian cancer samples of the mucinous type stained positive in only 56.5% of the cases, whereas benign Brenner tumors were positive in 20% of the cases (Fig. 3d).

**Fig. 4:**
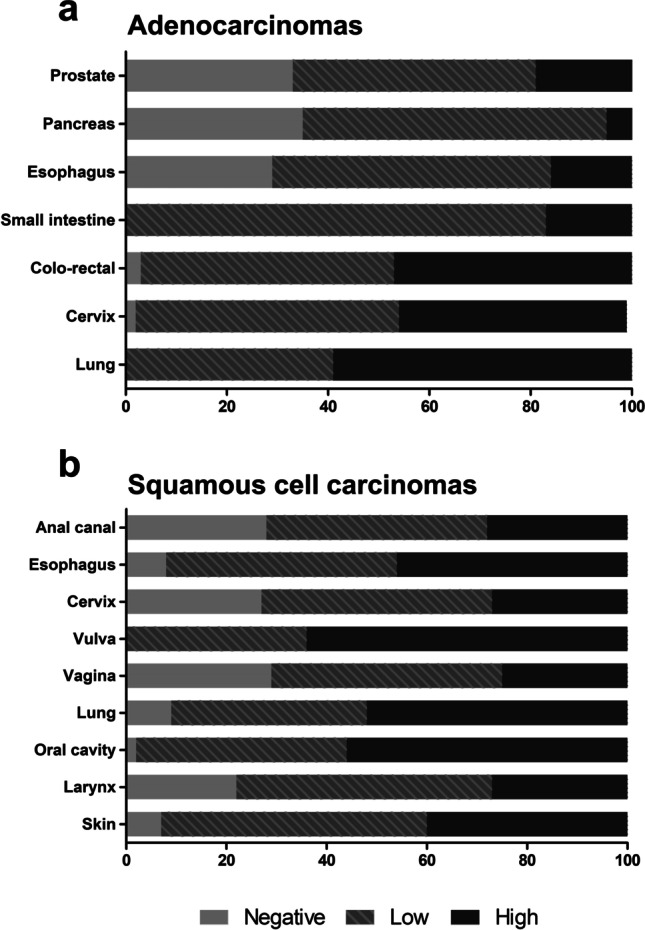
Percentage of YKL-40 positive tumor samples of all tumor samples in **a** adenocarcinomas and **b** squamous cell carcinomas on multi-tumor TMA

When comparing adenocarcinomas with squamous cell carcinomas, no general distinguishing pattern of YKL-40 expression was observed between these two entities (Fig. 4). While in esophageal cancer, for example, YKL-40 expression was found to be linked to the squamous cell cancer subtype (high expression in 46.1% of samples versus 16.1% in adenocarcinomas, Fig. 3e). In cervical cancer specimens, however, YKL-40 was found to be linked to the adenocarcinoma subtype (high YKL-40 expression in 45.5% of samples versus 27% in squamous cell carcinomas, Fig. 3f).

### YKL-40 protein expression in xenograft tumors of human cancer cell lines

**Fig. 5:**
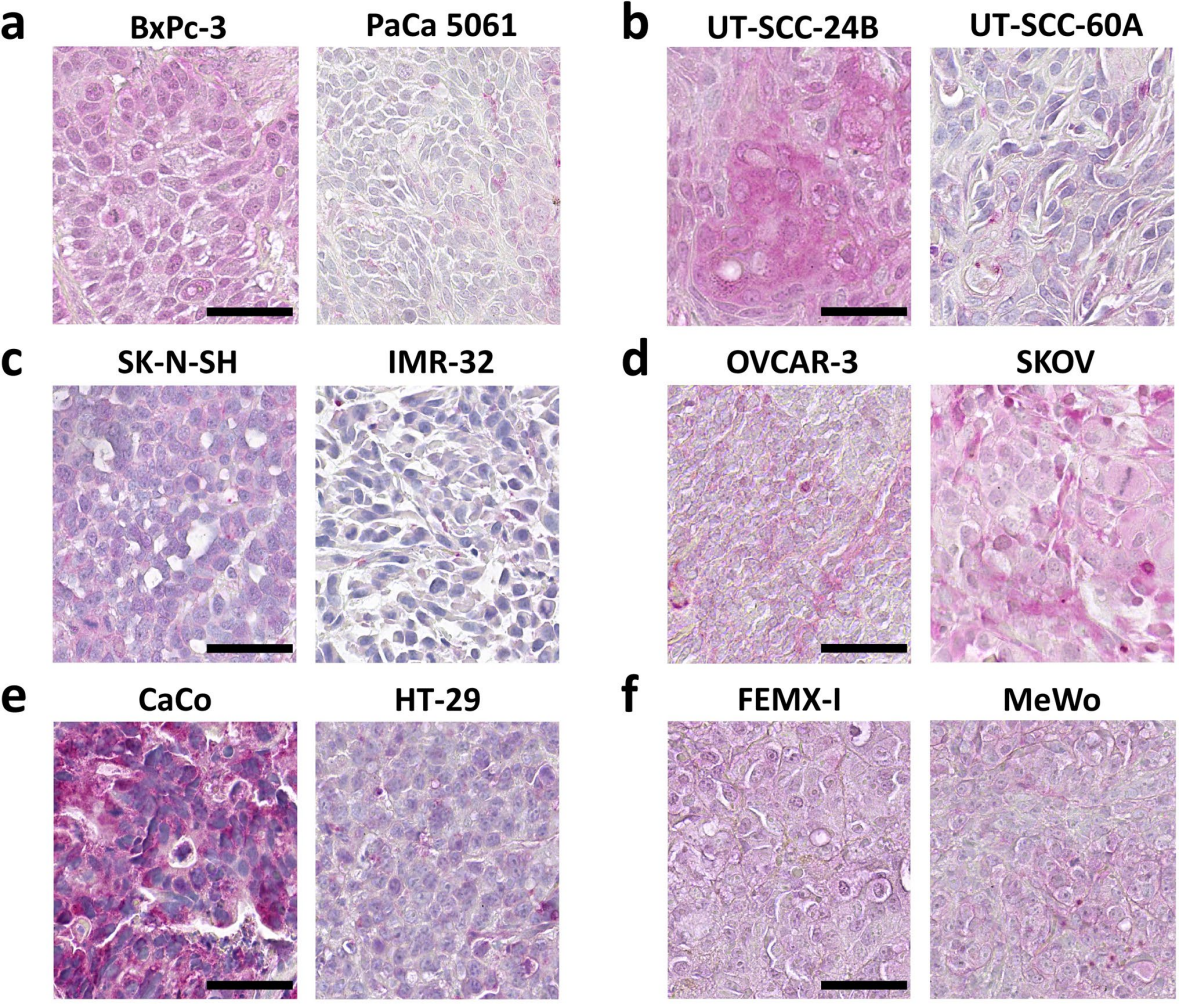
Representative YKL-40 immunostaining results in mouse xenograft tumors derived from different human cancer cell lines. Cancer cells were identified morphologically, and YKL-40 staining was assessed in areas of vital tumor tissue. **a** Pancreatic cancer cell lines, **b** head and neck squamous cell cancer cell lines, **c** neuroblastoma cell lines, **d** ovarian cancer cell lines, **e** colorectal cancer cell lines, **f** melanoma cell lines. Scale bars = 50 µm

**Fig. 6:**
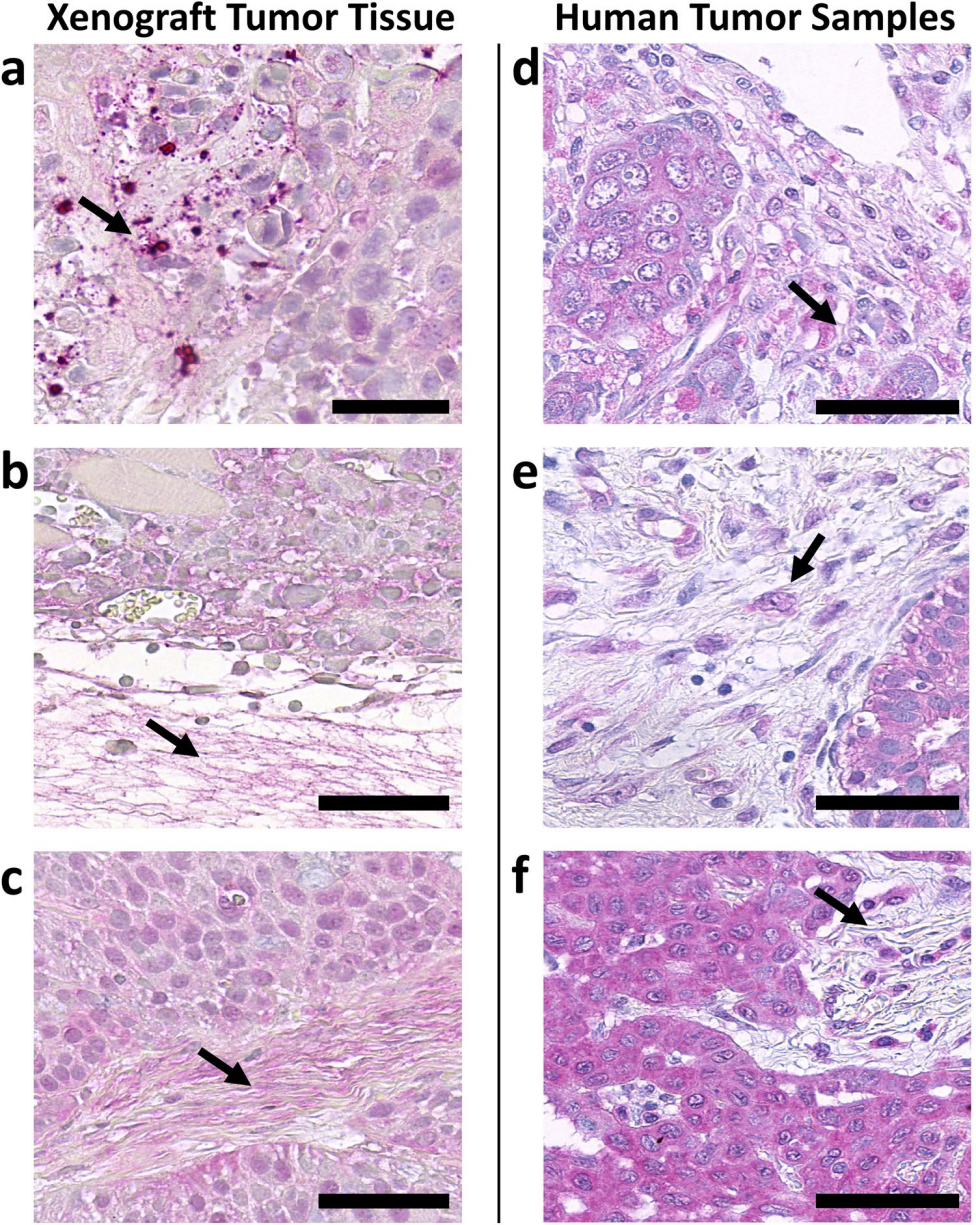
Representative YKL-40 immunostaining results of adjacent stroma in mouse xenograft tumor tissues **(a–c)** and human tumor samples **(d–f)**. Extracellular matrix components and stroma cells were identified morphologically. **a** Head and neck squamous cell cancer cell line UT-SCC-16A, **b** melanoma cell line FEMX-1, **c** pancreatic cancer cell line BxPC-3, **d** breast cancer, phyllodes subtype, **e** squamous cell esophageal cancer, **f** squamous cell lung cancer. Arrowheads point to fine granular staining in extracellular matrix components (mouse xenograft tumor tissue, **a–c**) or small nucleated cells residing in adjacent tumor stroma and surrounded by fine granular staining (human tumor samples, **d–f**). Scale bars = 50 µm

Tumor models, especially cancer cell-derived mouse xenografts, are increasingly used in cancer research. However, protein expression levels may differ significantly from clinical patient tumor samples to those grown in cell culture or as xenograft primary tumors in mice. Therefore, we assessed YKL-40 protein expression in human cancer cell lines directly taken from 2D-culture and paired mouse xenograft tumors. Cell pellets were embedded in agar and routinely processed as FFPE blocks, so that from a methodological point of view they were treated in the same way as primary biopsy specimens. We found that YKL-40 expression was immunohistochemically undetectable in all cancer cells grown in vitro except in SW480, CaCo2 and OVCAR3 cells (data not shown). Interestingly, YKL-40 expression became frequently positive under in vivo conditions in mouse xenograft tumors, especially in melanoma, pancreatic and colorectal cancer models (Table 2, Fig. 5). Moreover, our data suggest that human YKL-40 was released from tumor cells into murine extracellular matrix components (Fig. 6a-c).

**Table 2:** Immunohistochemical scoring of YKL-40 expression in xenografts of human cancer cell lines Staining intensity was assessed on a scale from ‘-’ = negative over ‘ + ’ = weak to ‘ +  + ’ = high expression

Cell Line	Score	Cell Line	Score
Melanoma		HNSCC	
LOX-IMVI	+	UT-SCC-2	-
FEMX-I	+ +	UT-SCC-16A	+
MV3	-	UT-SCC-24A	-
MeWo	+	UT-SCC-24B	+ +
MDA-MB-435	+	UT-SCC-60A	+
		Carey 24	-
Pancreatic Cancer			
PANC-1	+ +	Neuroblastoma	
PaCa 5061	+	KELLY	+
PaCa 5072	-	IMR-32	-
BxPC-3	+ +	LA–N-1	-
		LA–N-5	-
Colorectal Cancer		SK-N-SH	+
HT-29	+	LS	-
SW480	+		
CaCo	+ +	Prostate	
HCT 116	+	PC3	+
		C5	+
SCLC		DU-145	-
H69AR1	-	LNCaP	-
H69AR3	-		
NCI-H82	-	Osteosarcoma	
SW2	-	U2OS	+
		HOS	-
Breast Cancer			
DU4475	-	Ovarian Cancer	
MCF-7	+	OVCAR	+
MDA-MB-231	+	SKOV3	+ +
T-47D	-

### YKL-40 expression analysis in publicly available datasets

First, we checked the Broad Institute Cancer Cell Line Encyclopedia (CCLE) online tool for YKL-40 mRNA expression [38, 39]. We found that in only in 19% of 1,019 cell lines with expression values for YKL-40, expression z-scores were confidently above zero. Furthermore, we accessed publicly available GEO dataset GSE48433 comprising mRNA expression profiles of 49 human cancer cell lines and xenograft tumor fragments at passages 1, 4 and 10 from the Developmental Therapeutics Program of the National Cancer Institute (NCI DTP) [40]. In 33 out of 49 cell line/xenograft pairs, YKL-40 expression increased under in vivo conditions (Supplementary Fig. S4).

As the increased YKL-40 expression in vivo may result from interactions of cancer cells with their surrounding extracellular matrices, we searched the NCBI GEO database for expression data comparing cells mono-cultured and co-cultured with stromal cells. Only a limited number of experiments with YKL-40 expression data was available (GSE115052, GSE60035, GSE98154, GSE109577) [41–44]. A clear tendency of increased YKL-40 expression in co-culture could be observed in prostate and ovarian cancer cells (Supplementary Fig. S3).

## Discussion

Elevated YKL-40 serum levels have been described in a growing number of inflammatory and malignant diseases and for many of them, YKL-40 has been proposed as a prognostic biomarker [45]. The biological role and function of YKL-40 in malignant progression, however, remains elusive for multiple reasons. First, cellular receptors for YKL-40 have not yet been identified. Secondly, there is opposing data for the role of YKL-40 on proliferation and tumor growth in xenograft models [22, 23]. Thirdly, the question of whether serum YKL-40 in tumor patients is derived from production and release by the tumor cells themselves or from immune and/or stromal cells due to the tumor's immunologic reaction is a matter of debate [10].

This work complements a former immunohistochemical study on YKL-40 protein expression in normal adult human tissues by Ringsholt et al. [46]. The authors found YKL-40 to be an omnipresent protein throughout the normal tissues analyzed, but the number of samples was relatively small. Protein expression data in tumor tissues, however, are only available for some selected entities and subtypes. So far, integrative analyses of YKL-40 expression in cancer, whether in primary human tumor tissues or human cancer cell lines, are only available on the mRNA level. The lack of actual protein expression data is critical for the interpretation of the functional role of YKL-40 in tissues. In a comprehensive analysis of YKL-40 expression in differentiating mesenchymal stem cells (MSCs), Hoover et al. found undifferentiated MSCs to transcribe significant levels of YKL-40 mRNA, whereas YKL-40 protein was absent both in cell lysates and media supernatants [47]. Moreover, they found that YKL-40 mRNA was not immediately translated into protein, but with a delay of at least 24 h following the addition of osteoblast or chondrocyte differentiation media. The authors hypothesized that YKL-40 may be regulated by (one or more) microRNAs (miRNAs) binding to YKL-40 mRNA. This hypothesis was at least partly supported by association studies of miRNAs and mesenchymal markers (among them YKL-40) in glioblastoma [48], but needs further verification. These findings imply that mRNA quantification alone may not reflect the complex regulation of YKL-40 in cancer progression and provides a rationale for studying YKL-40 expression at the protein level.

In accordance with immunohistochemical data on normal tissues [46], it is not surprising that YKL-40 protein could be detected in a high number of tumors from all entities in our study. On the other hand, in a significant proportion of tumors YKL-40 expression was specifically absent, whereas in many tumors the staining frequency and intensity of stroma cells surpassed those of the tumor cells. We detected intensely stained stroma cells that were surrounded by finely granular material. Morphologically, these cells most likely represent infiltrating neutrophilic granulocytes. While Ringsholt et al. found that this observation was exceptionally made in surrounding tissues of the appendix [46], we found that this observation was true for most of the tumors. To what extent YKL-40 serum levels are fueled by release from the tumor cells themselves or from specific granules of the neutrophilic granulocytes [4] needs to be addressed in future studies. Further, YKL-40 expression in serum, tumor tissue and adjacent stroma should be studied coherently to answer the question of whether those patients with no YKL-40 expression in the tumor tissues still have elevated serum levels.

The herein used antibody Mab 201.F9 has been validated for immunohistochemistry purposes by others in the past and was demonstrated to have specific, strong labeling properties with a lack of nonspecific background staining [21, 49]. Moreover, we found that Mab 201.F9 showed no cross-reactivity with murine YKL-40. This notion is of specific importance when interpreting our results in mouse xenograft tumor tissues derived from human cancer cell lines. Our results clearly suggest that human cancer cell lines, when exposed to in vivo conditions, upregulate YKL-40 expression and secrete YKL-40 into the surrounding mouse-derived stroma. It is known that three-dimensional cell-to-cell contact and cell-to-matrix contact may influence a cell's gene expression pattern. Our findings were further corroborated by in silico analysis of YKL-40 mRNA expression profiles in murine tumor xenograft tissues compared with its expression in the parental cell line taken from 2-D culture.

In a glioblastoma xenograft model, Francescone et al. showed that YKL-40 binds to heparan sulfate of the ectodomains of syndecan-1 and initiates coupling with integrin α_v_β_3_ [17]. In endothelial cells this action was followed by activation of a signaling cascade engaging focal adhesion kinase (FAK) and the mitogen-activated protein kinase/extracellular signal-regulated kinase (MAPK/ERK) pathway leading to elevated levels of vascular endothelial growth factor (VEGF) and enhanced angiogenesis [17, 18, 22]. Syndecans act as matrix co-receptors with integrins, thereby mediating cell–cell and cell–matrix adhesion and promoting proliferation, differentiation and migration [50]. Upregulation of YKL-40 in vivo may be one essential component in the binding and activation of these receptors. In many of the cell lines tested in our study, YKL-40 mRNA expression increased markedly in vivo in the xenograft tumor tissues. These results may have implications for research on the role of YKL-40 in tumor models.

In the past, much of the work on the effect of YKL-40 on malignant progression in tumor models has been based on glioblastoma cells. This restriction is most probably due to the fact that only a few cell lines produce YKL-40 protein in vitro and in vivo such as the glioblastoma cell lines U87, U1242MG, U343MG and U1231MG, and the osteosarcoma cell line MG63 [51]. As in vitro analyses revealed lack of endogenous YKL-40 expression in other cancer cell lines, this notion provided reason to use them as the null background against which biological activities of YKL-40 have been examined, among them MDA-MB-231 and HCT-116 cells engineered to ectopically express YKL-40 [18]. Our results suggest that this ectopic overexpression of YKL-40 may override the pathways that physiologically (up-)regulate its expression in vivo. Therefore, careful consideration should be taken when choosing a tumor model. Our study may pave the way for examining the effects of YKL-40 in tumor models of other entities as well.

In conclusion, our data provide new insight into YKL-40 expression at the protein level in various tumor entities and its regulation in tumor models. Our results may provide a rationale for characterizing YKL-40 as a feasible distinguishing tissue marker in tumor subtypes. Our data also suggest that YKL-40 expression is a common feature of tumor progression in vivo, while the absence of YKL-40 expression in vitro may be an artifact of cell culture.

## Supplementary Information

Below is the link to the electronic supplementary material.

Supplementary file1 (PDF 1270 kb)

## Data Availability

All data are available in the manuscript.
