# Supplementary material for: YKL-40 protein expression in human tumor samples and human tumor cell line xenografts: implications for its use in tumor models
Source: Cell Oncol (Dordr). 2021 Aug 25;44(5):1183–95. doi: 10.1007/s13402-021-00630-z (PMC8516773; doi:10.1007/s13402-021-00630-z)
Supplement: Supplementary file 1 — Supplementary file1 (PDF 1270 kb) [file 13402_2021_630_MOESM1_ESM.pdf]

Supplemental Material

YKL-40 Protein Expression in Human Tumor Samples and Human Tumor Cell Line Xenografts: Implications for its use in tumor models

Lukas Clemens Böckelmann [1,2,*], Theresa Felix [1], Simona Calabró [1] and Udo Schumacher [1]

[1] Institute of Anatomy and Experimental Morphology, Center for Experimental Medicine, University Cancer Center Hamburg, University Medical Center Hamburg-Eppendorf, Hamburg, Germany

[2] Department of Oncology, Hematology and Bone Marrow Transplantation with section Pneumology, University Cancer Center Hamburg, University Medical Center Hamburg-Eppendorf, Hamburg, Germany

[*] Correspondence: l.boeckelmann@uke.de; Phone +49 40 7410 20978; Fax +49 40 7410 55427

Figure S1: Monoclonal antibody Mab 201.F9 specifically binds human YKL-40 but not murine YKL-40 in immunohistochemistry of FFPE tissue sections. (A) Human brain, (B) murine brain, (C) human kidney, (D) murine kidney, (E) human liver, (F) murine liver. ISO = Isotype antibody control. Scale bars = 100 µm.

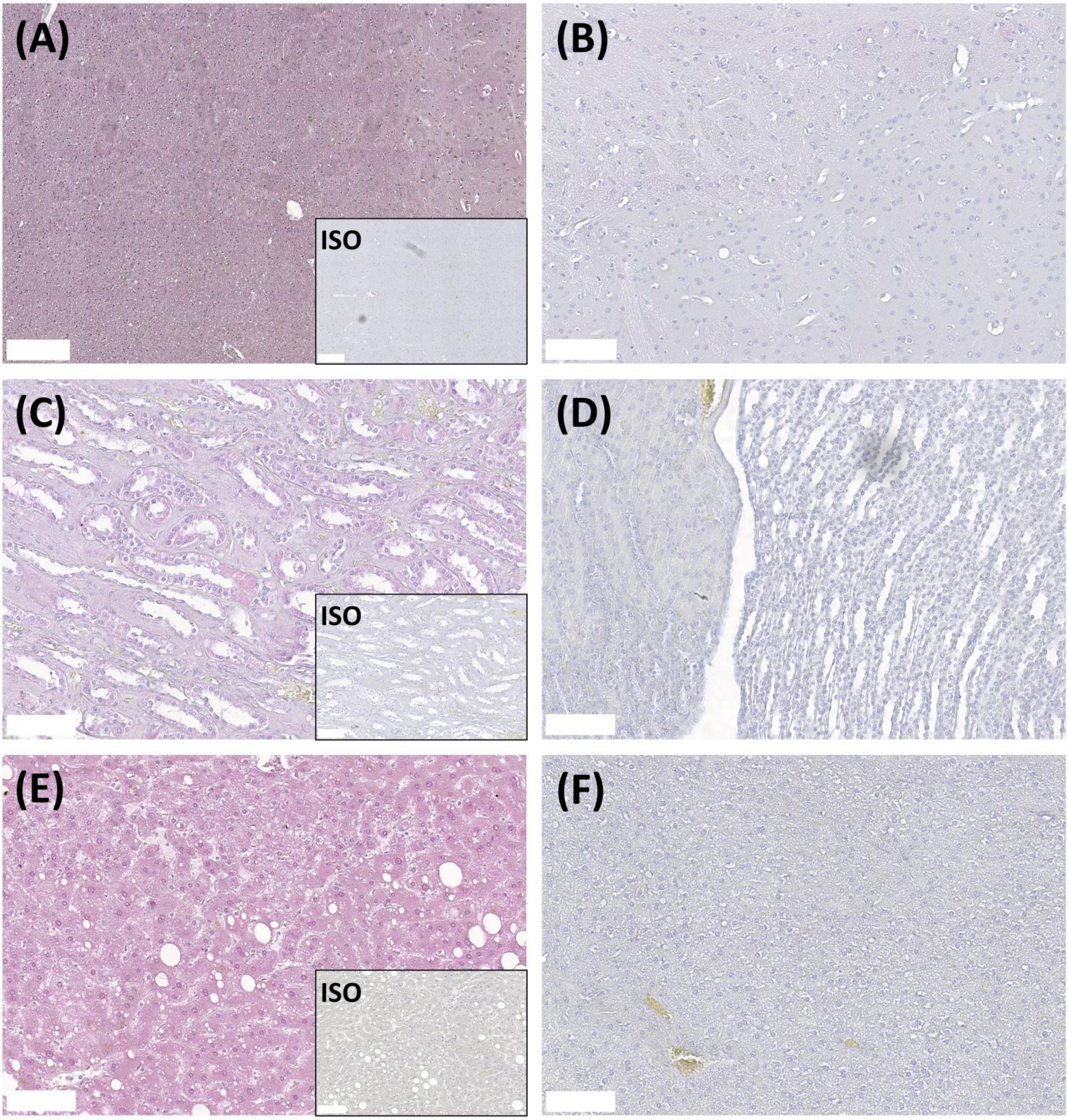

Figure S2: Immunohistochemical staining of human colon tissue with anti YKL-40 antibody MAb 201.F9. (A) Positive control, (B) isotype control antibody, and (C) absorbed with recombinant human YKL-40 protein.

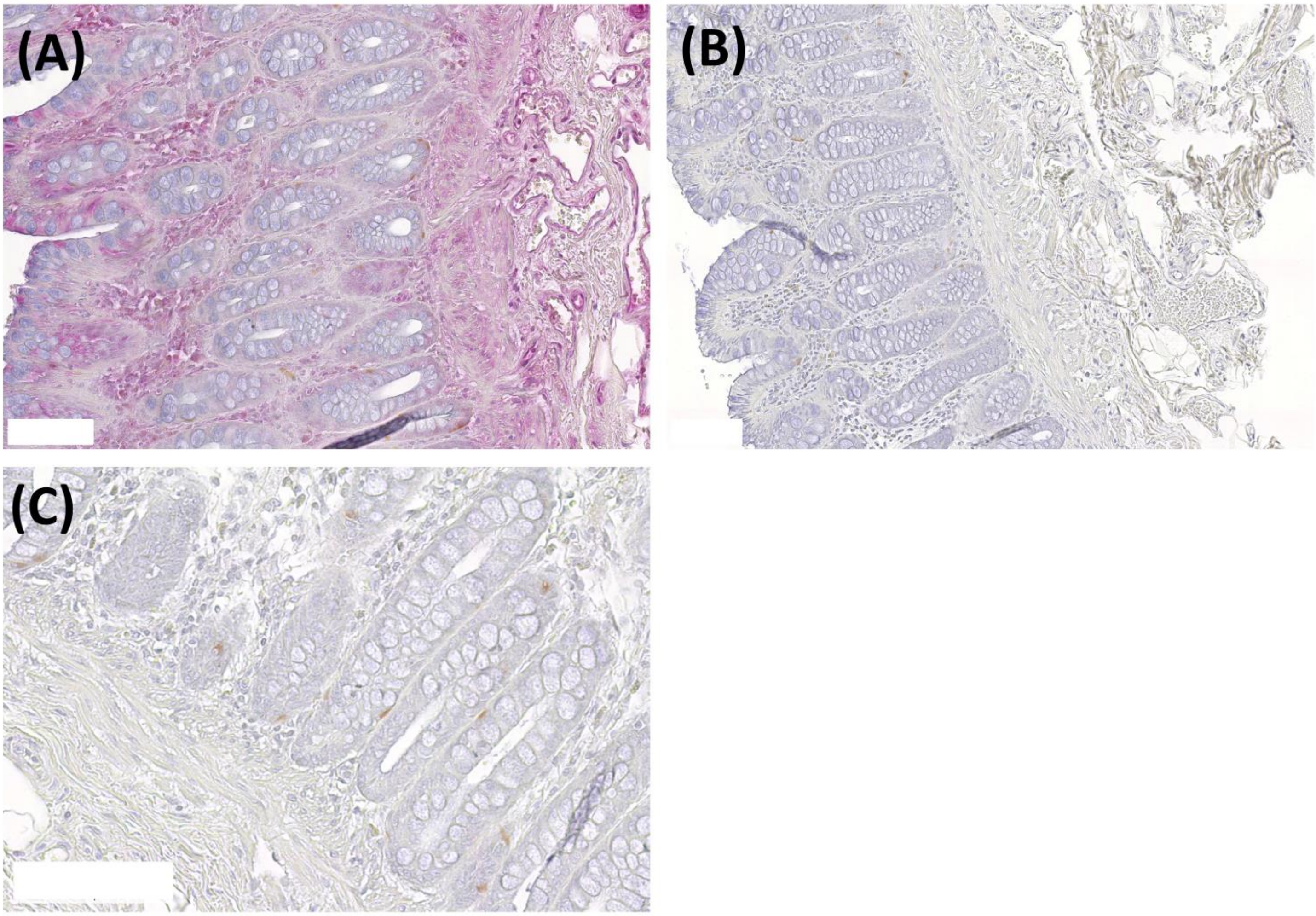

Figure S3: Analysis of CHI3L1 (YKL-40) mRNA expression profiles in publicly available GEO datasets. (A) Primary prostate organoid transcriptomic profile in co-culture with primary prostate stroma [1], (B) interaction between OVCAR-3 cells and mesenchymal stroma cells [2], (C) SKOV3 cells that could form heterotypic spheroids with high grade serous ovarian cancer derived cancer-associated fibroblasts and those remaining individual SKOV3 cells [3], (D) tissue-engineered mimic of ovarian cancer microenvironment [4].

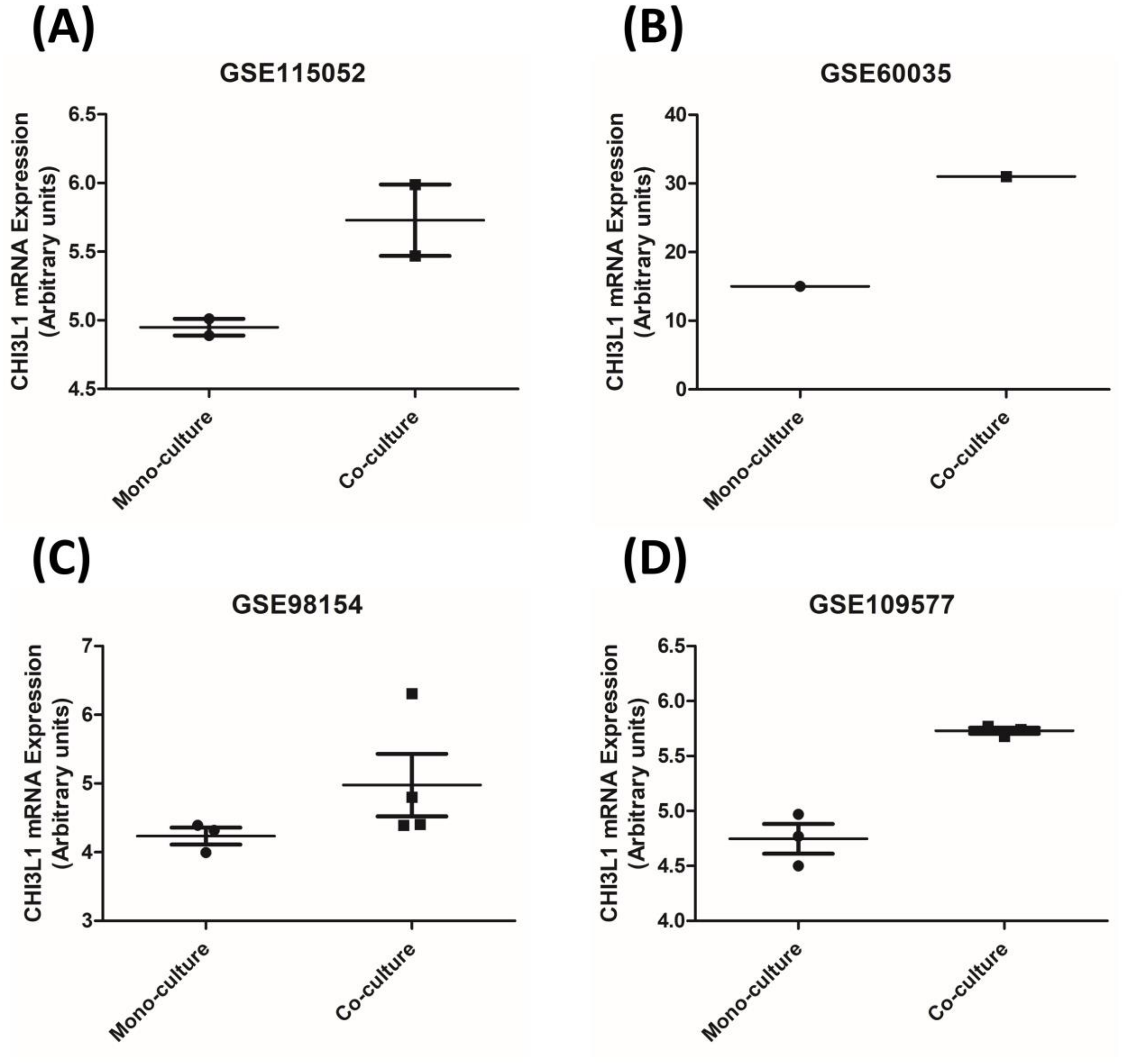

Figure S4: Analysis of CHI3L1 (YKL-40) mRNA expression profiles in publicly available GEO dataset GSE48433. Data derived from microarray analysis of xenograft models in use at the Developmental Therapeutics Program of the National Cancer Institute (DTP-NCI) [5]. For originating cell lines (passage 0, P0) and xenograft tumor fragments at passages 1, 4, and 10 (P1, P4 and P10), RNA was isolated, cDNA transcribed and hybridized to Affymetrix HG-U133 Plus 2.0 arrays. P0 samples have 2-3 replicates, whereas P1, P4 and P10 samples have 5 replicates. This dataset comprises a total of 823 array files.

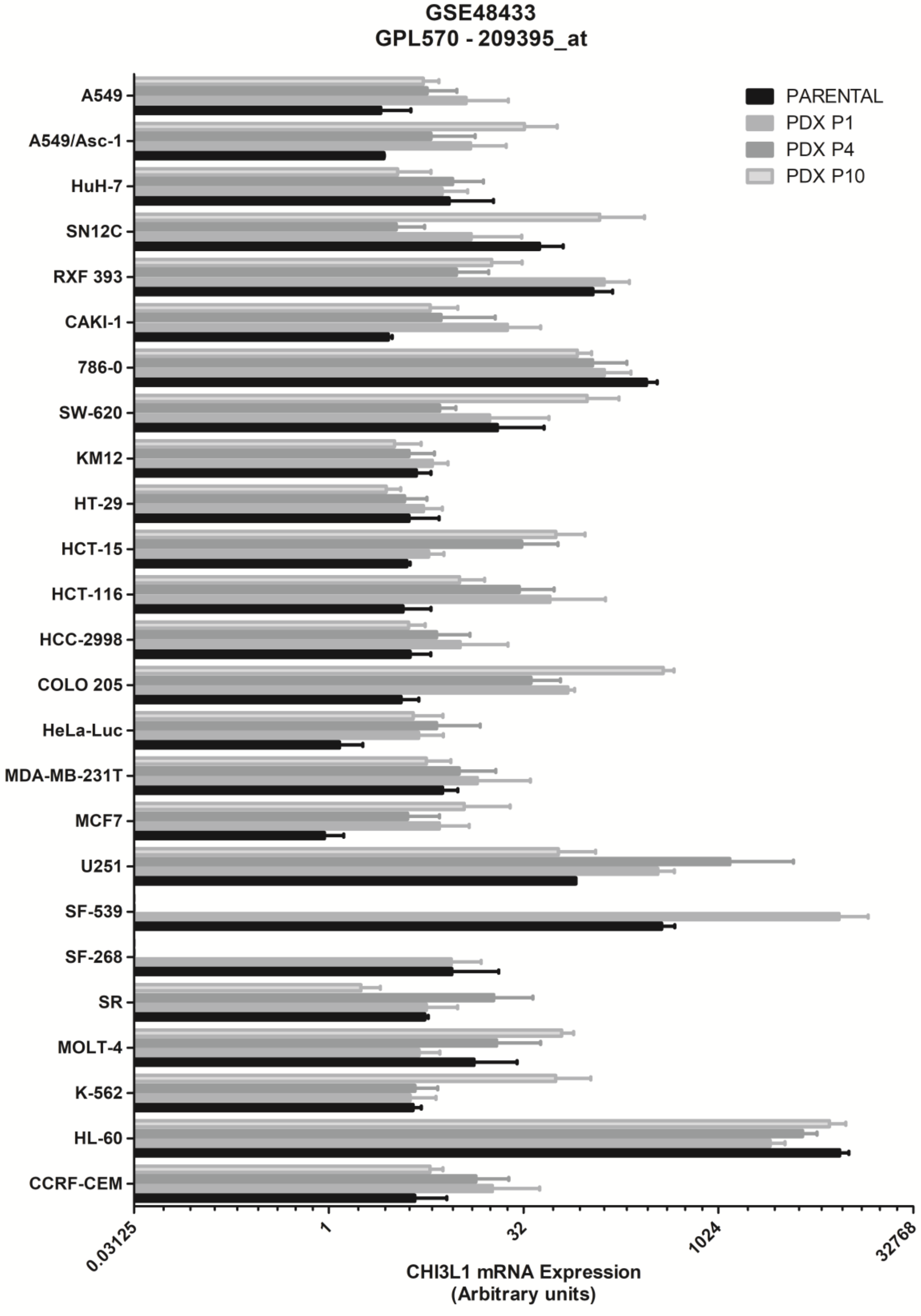

Figure S4 continued

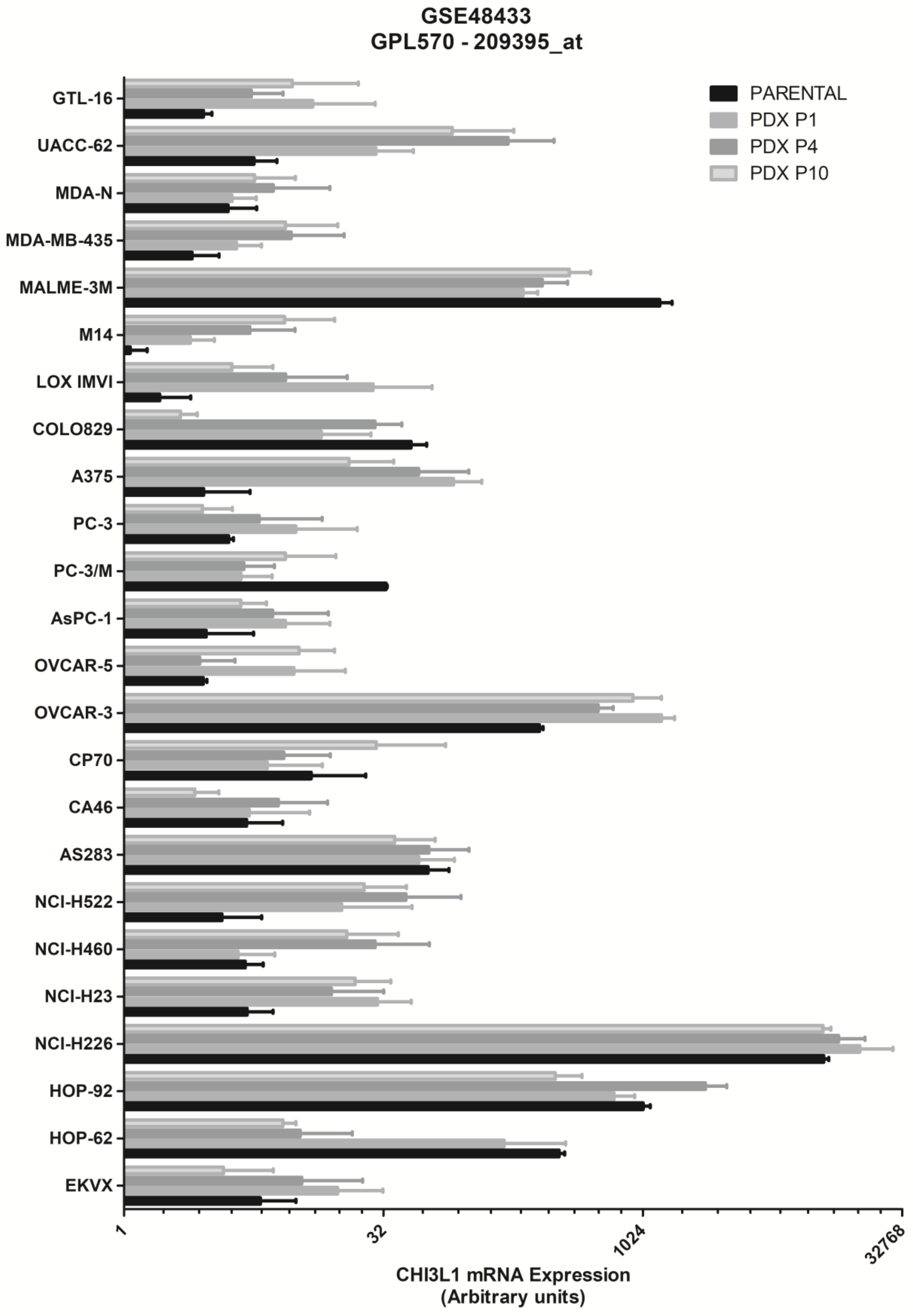

References

1. Z. Richards, T. McCray, J. Marsili, M. L. Zenner, J. T. Manlucu, J. Garcia, A. Kajdacsy-Balla, M. Murray, C. Voisine, A. B. Murphy, S. A. Abdulkadir, G. S. Prins, und L. Nonn, Prostate Stroma Increases the Viability and Maintains the Branching Phenotype of Human Prostate Organoids. IScience **12**, 304–317 (2019).

2. Y. Yang, A. Otte, und R. Hass, Human Mesenchymal Stroma/Stem Cells Exchange Membrane Proteins and Alter Functionality During Interaction with Different Tumor Cell Lines. Stem Cells and Development **24**, 1205–1222 (2014).

3. Q. Gao, Z. Yang, S. Xu, X. Li, X. Yang, P. Jin, Y. Liu, X. Zhou, T. Zhang, C. Gong, X. Wei, D. Liu, C. Sun, G. Chen, J. Hu, L. Meng, J. Zhou, K. Sawada, R. Fruscio, T. W. Grunt, J. Wischhusen, V. M. Vargas-Hernández, B. Pothuri, und R. L. Coleman, Heterotypic CAF-tumor spheroids promote early peritoneal metastasis of ovarian cancer. J Exp Med **216**, 688–703 (2019).

4. D. Loessner, A. Rockstroh, A. Shokoohmand, B. M. Holzapfel, F. Wagner, J. Baldwin, M. Boxberg, B. Schmalfeldt, E. Lengyel, J. A. Clements, und D. W. Hutmacher, A 3D tumor microenvironment regulates cell proliferation, peritoneal growth and expression patterns. Biomaterials **190–191**, 63–75 (2019).

5. M. G. Hollingshead, L. H. Stockwin, S. Y. Alcoser, D. L. Newton, B. C. Orsburn, C. A. Bonomi, S. D. Borgel, R. Divelbiss, K. M. Dougherty, E. J. Hager, S. L. Holbeck, G. Kaur, D. J. Kimmel, M. W. Kunkel, A. Millione, M. E. Mullendore, H. Stotler, und J. Collins, Gene expression profiling of 49 human tumor xenografts from in vitro culture through multiple in vivo passages - strategies for data mining in support of therapeutic studies. BMC Genomics **15**, 393 (2014).
